# Development and psychometric validation of the Sexual Health Literacy Measurement Tool for Undergraduate Students (SHL-US) in Thailand

**DOI:** 10.1371/journal.pone.0359881

**Published:** 2026-10-09

**Authors:** Natnapa Heebkaew Padchasuwan, Jintapa Benchamas, Pokkamol Laoraksawong, Nopparat Senahad, Pannee Banchonhattakit, Surachai Phimha

**Affiliations:** 1 Faculty of Public Health, Khon Kaen University, Khon Kaen, Thailand; 2 Faculty of Public Health, Valaya Alongkorn Rajabhat University under the Royal Patronage, Pathum Thani, Thailand; Commonwealth Scientific and Industrial Research Organisation, AUSTRALIA

## Abstract

**Background:**

Sexual health literacy refers to an individual’s capacity and proficiency in managing sexual health. Currently, there are limited instruments available to assess sexual health literacy among undergraduate students, particularly in Thailand. Thus, the current study aimed to develop and psychometrically validate the sexual health literacy measurement tool for undergraduate students (SHL-US) in Thailand.

**Methods:**

The study involved 1,235 undergraduate students from public and private universities in Thailand. A multi-stage probability sampling design was used, with simple random selection of institutions (one public and one private university per region, across four regions), faculties, and classrooms, followed by complete enumeration of eligible, consenting students within the selected classrooms. A pilot test (n = 50) and exploratory factor analysis (n = 300) preceded the confirmatory sample (n = 1,235). Thirteen experts examined the content validity. The quality of the instrument was tested by examining its psychometric properties using exploratory and confirmatory factor analyses.

**Results:**

Forty-nine items entered the EFA; 47 were retained (4 factors, 53.17% variance explained). CFA removed two more low-loading items (<0.50), yielding the final 45-item, four-factor structure: access (12), understanding (12), appraisal (10), application (11). Additional internal consistency analysis yielded satisfactory results, with an overall Cronbach’s alpha coefficient of 0.976. The item-total correlation (ITC) was 0.284–0.755, and content validity, assessed via the content validity index (CVI), was high (CVI = 0.94). Confirmatory factor analysis, using the item set confirmed against the CFA diagram and retaining only correlated residuals with genuine item-content justification, supported an acceptable four-factor structure. The four domains were strongly inter-correlated, suggesting they are best interpreted and scored as facets of a single overarching SHL construct rather than fully independent subscales.

**Conclusion:**

The SHL-US (45 items) showed acceptable four-factor fit and good internal consistency, though the four domains behave as correlated facets of one SHL factor rather than separable competencies. Used as a single overall score, it shows potential for assessing sexual health literacy among Thai undergraduates.

## Introduction

Globalization and rapid technological change have made sexuality-related information widely available, and young people increasingly encounter confusing and conflicting messages about relationships and sex during the transition to adulthood [1]. In Thailand, sexual socialization within families varies significantly across the four regions, reflecting the country’s cultural diversity; although most parents report discussing sexuality with their adolescent children, nearly all do not perceive them as engaging in any sexual risk behaviour [2]. Evidence from Thai school settings suggests that the competencies needed to navigate this environment remain underdeveloped: most students report low to moderate levels of sexual health literacy and correspondingly low levels of preventive behaviour, and their main sources of sexual-health guidance are peers and the Internet rather than formal instruction. Notably, neither students nor teachers in that study were familiar with the concept of sexual health literacy [3].

Sexual health literacy is the ability and skills to take care of one’s sexual health, including accessing information, understanding, appraising, and applying that information to sexual healthcare, health promotion, and the correct prevention of risky sexual behaviours [4]. In the present study, sexual health literacy (SHL) is operationally defined as the cognitive and social competencies that enable an undergraduate student to access, understand, appraise, and apply information pertaining to sexual health [4,5], in order to promote sexual health and prevent risky sexual behaviours and their proximal consequences (STIs/HIV and sexually acquired unintended pregnancy) [6]. This definition deliberately bounds the construct. SHL is narrower than sexual and reproductive health literacy (SRHL), which additionally spans the reproductive continuum – fertility, life-course contraceptive decision-making, and antenatal and maternal care [7]; here, reproduction is engaged only insofar as pregnancy is a direct consequence of sexual behaviour. SHL is likewise distinct from sexuality education, the pedagogical process through which such literacy may be cultivated [1], rather than the individual-level competency measured in the present study [8]. To operationalise this construct, the present study adopts the structural domains of the integrated health literacy framework [4], focusing on individuals’ capabilities to access, understand, appraise, and apply sexual-health information in their own lives. Addressing SHL is consistent with the broader public health goal of tackling the social and structural root causes of poor sexual health outcomes including HIV, HSV-2 and other sexually transmitted infections, unwanted pregnancy, and reproductiveage maternal mortality in order to achieve fairer outcomes [9].

Despite this relevance, sexual health literacy among undergraduate students has received little attention. Existing instruments were developed for other populations and orient their content toward reproduction and pregnancy prevention rather than sexual health as a whole. The reproductive health literacy questionnaire for Chinese unmarried youth aged 15–24 operationalises literacy across the reproductive continuum and scores it as knowledge, using correct/incorrect and difficulty-rated items organised by health domain rather than by literacy competency [10]. The Sexual Health Literacy for Adults questionnaire (SHELA) was developed for ever-married Iranian adults aged 18 years and over, with a substantial share of items framed around the marital dyad, and was validated in a predominantly female sample (75.2% women) [11]. The sexual health literacy measurement tool for pregnancy prevention among adolescents (SHL-PPA) restricts its content to pregnancy prevention among Thai adolescents aged 13–19, excludes sexually transmitted infections, and yielded a three-factor solution in which understanding and appraisal merged into a single component [12]. Although each instrument reported acceptable content validity and internal consistency, their target populations, content boundaries and factor structures leave the sexual health literacy of undergraduate students unaddressed, and their pregnancy-centred framing foregrounds risks disproportionately attributed to young women. Intervention work with Thai undergraduates has followed a similar pattern: a health literacy based safe-sex programme improved health literacy and contraceptive practice though not intention but was confined to female students who had a boyfriend, recruited from two universities in a single province (n = 73), and targeted pregnancy prevention specifically [13]. Consequently, no instrument calibrated to the Thai undergraduate population as a whole is currently available, even though undergraduates are a crucial force in societal development and tend to participate in regional and global communities through social interactions and employment pursuits.

The four foundational competencies of the integrated health literacy model access, understanding, appraisal, and application [4] are adopted here as both necessary and sufficient to evaluate SHL. They are necessary because they form a sequential, non-redundant information-processing pathway: a student might access sexual health information but fail to comprehend it, or understand it but lack the critical capacity to appraise source credibility amid pervasive online misinformation [4,14]. Even accurate appraisal does not guarantee adaptive application, such as negotiating condom use or seeking clinical services; among Thai undergraduates, only the application domain remained independently associated with reduced risky sexual behaviour after adjustment for sociodemographic and lifestyle factors [15]. Omitting any single competency therefore severs the continuum from information acquisition to protective health behaviours. They are sufficient because Sørensen et al.‘s model [4] synthesizes 17 definitions and 12 conceptual frameworks into a cohesive, content-generic architecture: topic-specificity is driven by contextual item content (i.e., sexual health promotion and risk prevention) rather than by redundant competency domains. Earlier health literacy typologies are thus fully subsumed within these four pillars, with communicative and negotiation skills naturally embedded within the application domain.

Because these core domains are content-generic, they transcend specific sexual orientations and gender identities. Sexual behaviour and sexual risk are not reducible to identity labels, as sexual practices and partnerships frequently occur across identity categories a principle embedded in global HIV programming, where UNAIDS classifies key populations partly by sexual practice, including, in its own wording, “gay men and other men who have sex with men” a behavior-based rather than purely identity-based category [16], a pattern well documented among Thailand’s diverse gender and sexual minority groups [17]. Securing credible information, evaluating online misinformation, and navigating medical services represent universal mandates for sexual self-care, consistent with the World Health Organization’s inclusive conceptualization of sexual health as holistic well-being extending beyond exclusively heterosexual or reproductive frameworks [6]. Indeed, robust SHL is particularly crucial for LGBTQ+ populations, who face a disproportionate epidemiological burden of HIV and other sexually transmitted infections (STIs) in Thailand [18,19] and globally, while encountering systemic barriers to affirming healthcare driven by minority stress [20,21]. Therefore, validating this instrument within a sample comprising a substantial proportion of LGBTQ+ youth is integral, rather than peripheral, to the construct validity of SHL. The coverage of LGBTQ + -specific item content, and the fact that measurement invariance across gender and sexual-orientation groups was not tested in the present study, are addressed explicitly in the Limitations. We, therefore, aimed to develop a sexual health literacy assessment instrument and conceptual framework from an integrated model of health literacy [4]. An assessment of sexual health literacy among undergraduates would benefit relevant agencies in planning prevention, assistance, and management of issues affecting this demographic, including fostering positive sexual behaviors and enabling adaptability in sexual practices to mitigate potential adverse effects.

## Materials and methods

### Study population

This research entailed the creation of a questionnaire for undergraduates in Thailand and its validation through an examination of its psychometric properties. This study was approved by the Ethics Committee of Human Research at Khon Kaen University, Khon Kaen, Thailand (#HE662286) on 6 March 2024. This approval covered the broader project from which the present validation data were drawn [15].

### Study framework

**The four steps involved in developing the SHL are as follows**

#### Step 1: Item generation.

The primary concept delineating sexual health literacy, following the literature review, was an individual’s ability to seek, access, and comprehend health information; articulate, interpret, assess, and evaluate the information received; and communicate and utilize this information to make informed decisions regarding health promotion and disease prevention. We based our instrument development on the conceptualization [4]. The item pool was adapted exclusively from the 47-item European Health Literacy Survey Questionnaire (HLS-EU-Q47), which operationalizes the four competencies of the integrated model [22]. Rather than a verbatim translation of the source instrument, items were developed de novo in Thai: the wording of the HLS-EU-Q47 matrix was adapted and additional items were written to fit the sexual-health context of Thai undergraduate students across the two functional foci of sexual-health promotion and prevention of risky sexual behavior. No items were drawn from any other instrument. Because the items were newly generated rather than translated, a full forward- and back-translation procedure was not applicable; instead, conceptual and content equivalence between the source framework and the Thai items was established through procedures consistent with the expert-committee and pre-testing stages of the cross-cultural adaptation guideline [23], an approach aligned with the universalist model of equivalence, which requires conceptual equivalence to be established in the target culture before adaptation or de novo item development [24,25]. The resulting evidence of equivalence is reported in Steps 2 and 3 (expert panel, I-CVI and S-CVI; pilot testing) and in the factor-analytic results. The questionnaire comprised four components 1) access, 2) understanding, 3) appraisal, and 4) application which were proxies for sexual risk behavior prevention and sexual health literacy. A four-level Likert-type scale constituted the assessment format. The scores ranged from 1 to 4, where 1 represented Very Difficult, 2 Quite Difficult, 3 Easy, and 4 Very Easy. The four categories defining the criteria for interpreting the sexual health literacy scores were excellent (>84%), sufficient (67–84%), problematic (50–66%), and inadequate (<50%) [15]. Missing item-level responses were negligible and were not subject to any imputation or exclusion procedure.

#### Step 2: Item pool.

The researchers used the results from the first round to construct a questionnaire framework and drafted 50 closed-ended questions. Subsequently, the questionnaire was sent to 13 experts on sexual health literacy and health behaviors representing four regions in Thailand: 3 from the northern region, 3 from the central region, 3 from the southern region, and 4 from the northeastern region. The purpose was to review the consistency and appropriateness of the questions, clarity of the text, and comprehensiveness of the content, along with providing additional suggestions and recommendations, with each item rated on a scale from inconsistent to very consistent. The Content Validity Index (CVI) was calculated. The item-specific content validity index (I-CVI) ranged from 0.92 to 1.00, while the overall content validity index (S-CVI) was 0.94. We evaluated items having an I-CVI exceeding 0.90 and assessed the content validity of the whole instrument (S-CVI). The acquired value must be no less than 0.80, and items below this threshold or judged superfluous were removed. Of these, 49 were maintained, while one was deemed appropriate for removal.

#### Step 3: Pilot testing.

A pilot test of the instrument was conducted among 50 undergraduate students with characteristics similar to those of the target population. The reliability of the items was checked for internal consistency using Cronbach’s alpha coefficients. This research’s acceptance criterion was 0.7 or higher [26]. Following the pilot testing phase, an independent sample of 300 undergraduate students was recruited for exploratory factor analysis (EFA).

#### Step 4: Evaluation of psychometric properties.

Field testing was conducted to evaluate the psychometric properties of the instrument among 1,235 undergraduate students in Thailand, recruited as part of a larger project on sexual health literacy and risky sexual behaviour; findings on factors associated with risky sexual behaviour in a subsample of this cohort have been reported separately [15]. The present paper reports the development and full psychometric evaluation of the instrument. In this phase, the data were used to assess the construct validity of the SHL instrument using confirmatory factor analysis (CFA) based on the factor structure identified from the previous exploratory factor analysis (EFA) phase and reliability testing of the questionnaire items. The sexual health literacy measurement tool for undergraduate students (SHL-US) can be found in S1 Questionnaire.

The number of items retained and reasons for exclusion at each development stage are summarized in Fig 1.

**Fig 1:**
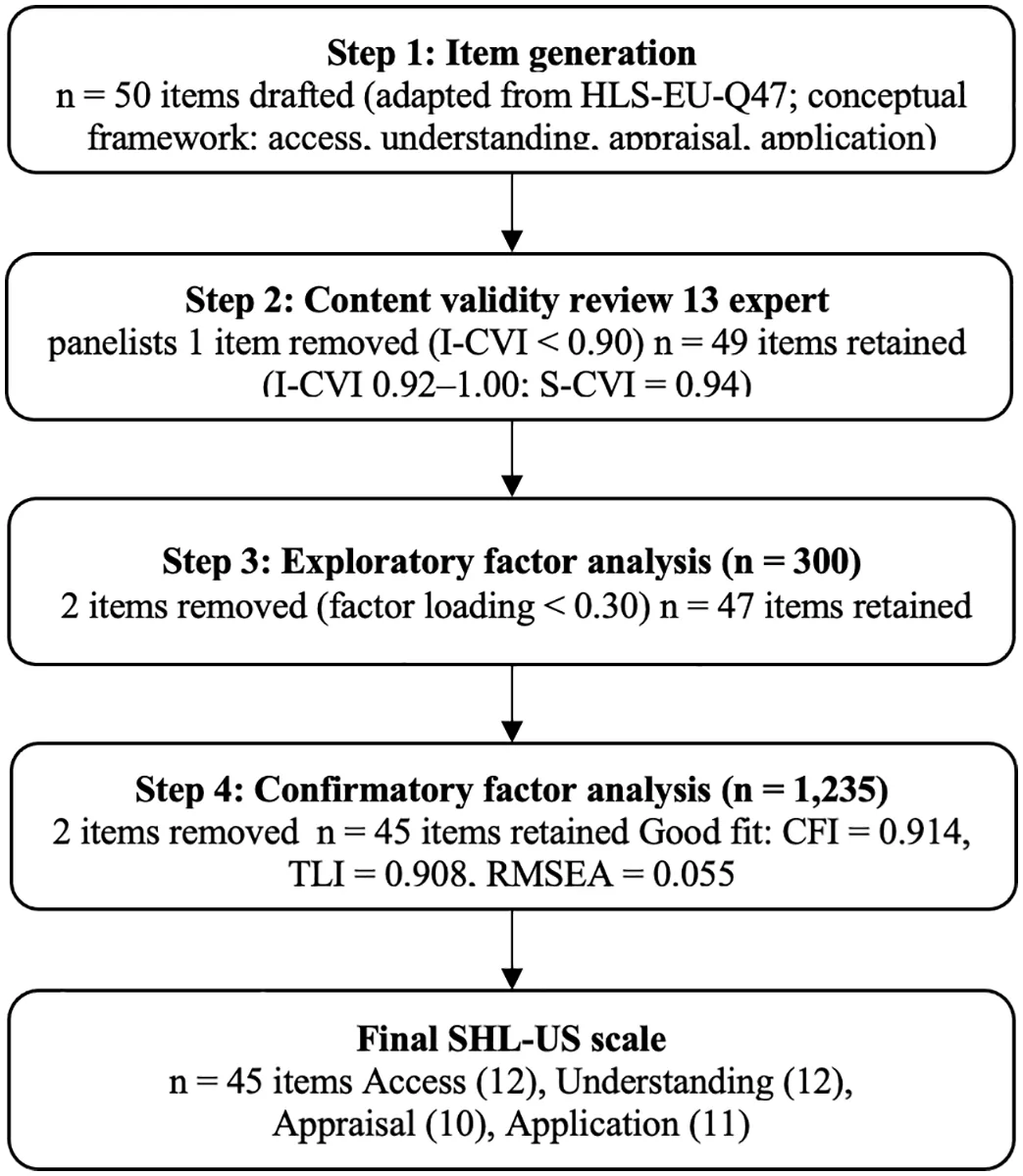
Item attrition flow diagram SHL-US development. Number of items retained and reasons for exclusion at each development stage: item generation (50 drafted), expert review (49 retained, 1 removed), pilot testing (n = 50), EFA (47 retained, 2 removed for loading < 0.30; n = 300), and CFA (45 retained, 2 removed for loading < 0.50; n = 1,235).

### Sample and data collection

The population comprised undergraduate students in Thailand’s universities, both public and private. The following inclusion criteria were used to select the sample: 1) no history of sexually transmitted diseases, 2) no history of unwanted pregnancy during the study period or in the past, and 3) willingness to participate in the study. The exclusion criteria were as follows: 1) people who were sick and 2) inability to participate in the survey and submit information. Students with a history of STI or pregnancy were excluded so that the instrument was calibrated on the primary-prevention target population undergraduates who had not yet experienced these adverse outcomes and to reduce reverse causation and confounding in the associated risk-factor analysis reported separately [15], in which prior events may alter both current sexual health literacy and current behavior. Validity is a property of a specified population and intended use rather than of an instrument in the abstract [27]. A multi-stage probability sampling design was used, with simple random selection of institutions (one public and one private university per region, across four regions), faculties, and classrooms, followed by complete enumeration of eligible, consenting students within the selected classrooms. Sampling proceeded in five stages, with randomization at three successive levels. Thailand’s four regions (central, northern, northeastern, and southern) served as sampling strata (Stage 1). Within each region, institutions were stratified into public and private types, and one institution of each type was drawn by simple random sampling (lottery), yielding eight institutions (Stage 2). Within each selected institution, three faculties were drawn by simple random sampling (Stage 3), and within each faculty five classrooms were drawn by simple random sampling, covering all year levels (Stage 4). All students in the selected classrooms who met the inclusion criteria were then invited to participate, and those who consented and completed the questionnaire constituted the analytic sample (Stage 5). Sampling units at each randomized stage therefore had a known, non-zero probability of selection; the final stage was a complete enumeration within the sampled classroom clusters rather than convenience recruitment, although participation within those clusters remained voluntary. The sample size was calculated using the formula for multiple logistic regression analysis [28], adjusting for the influence of relationships with the variance inflation factor (VIF). The researcher selected a correlation coefficient (ρ) of 0.8. The sample size was then adjusted for a 10% dropout rate using the formula to determine the sample size [29], resulting in a sample size of 1,235 people. Additionally, as this study involved exploratory and confirmatory factor analysis, the sample size was also evaluated against established guidelines for factor-analytic studies. The subjects-to-item ratio was approximately 25:1 (1,235 participants relative to the 49-item pool entered into EFA), which substantially exceeds the minimum recommended ratio of 10:1 [30]. Recruitment ran from 15/03/2024 to 31/05/2024, satisfying the sample size adequacy criteria for stable factor solutions proposed by MacCallum et al. [31]. After each class session, a researcher not the course instructor introduced the study and distributed a written information sheet covering its objectives, voluntary and anonymous participation, potential discomfort, confidentiality, and contact information. All participants were adults aged 18 or older. As the questionnaire was anonymous and collected no identifying information, consent was obtained on an opt-out basis: returning the completed questionnaire via a collection box constituted consent, while non-return unobservable to the researcher or instructor constituted non-participation. No incentives were offered. This procedure, including implied consent for an anonymous survey, was approved by the Research Ethics Committee. No names, student IDs, or other identifiers were recorded; questionnaires were returned to a sealed box, stored securely with access restricted to the research team, and reported only in aggregate. Several features also reduced social desirability bias in reporting sensitive sexual-health information: self-administration, anonymity, data collection by a non-authority researcher, unobservable non-participation, and no incentives. Anonymous, self-administered modes are associated with more candid reporting of sensitive sexual behavior than interviewer-administered modes, though they do not eliminate such bias [32]. The 25:1 ratio describes the confirmatory sample (n = 1,235) relative to the 45/49-item pool; the exploratory factor analysis was conducted on a separate pilot sample of n = 300 (see EFA Results), giving an EFA specific subjects to item ratio of 300:49 ≈ 6.1:1.

### Data analysis

Statistical analyses were carried out using IBM SPSS 26.0, with confirmatory factor analysis performed using its AMOS module. The structural validity and reliability of sexual health literacy were examined using exploratory factor analysis (EFA) in SPSS. Principal axis factoring (PAF) was used as the extraction method, with Promax and internal consistency, respectively. Kaiser-Meyer-Olkin (KMO) and Bartlett’s Tests of Sphericity were used to determine the appropriateness of the samples for factor analysis. Eigenvalues above 1 and factor loadings ≥0.30 were used as the retention/inclusion criteria, consistent with common statistical-software defaults; more rigorous alternatives such as parallel analysis have since been recommended in the literature [33]. Confirmatory factor analysis was performed, and four dimensions (accessing, understanding, appraisal, and applying) were specified. Several goodness-of-fit indicators, including Relative Chi-square (χ^2^/df < 3), Goodness of Fit Index (GFI > 0.90), Adjusted Goodness of Fit Index (AGFI > 0.90), the Root Mean Square Error of Approximation (RMSEA< 0.06), Root of Mean Square Residual (RMR < 0.05), Normed Fit Index (NFI > 0.90), Tucker-Lewis Index (TLI > 0.95), and Comparative Fit Index (CFI > 0.95) were selected for reporting the analysis outcomes [34,35]. Modification indices (MIs) were examined only after estimation of the initial CFA model. Error covariances were added only when supported by both substantial modification indices and theoretical justification [36,37]. Convergent validity is based on standardized factor loadings (λ > 0.50), composite reliability (CR > 0.70), and average variance extracted (AVE > 0.50) [38]. Finally, Cronbach’s alpha coefficient (at an acceptable level of 0.70) for each dimension and the whole scale was calculated so as to examine the internal consistency and item-total correlation above 0.300 [26,39]. On the error-covariance criterion: of the 25 residual pairs flagged by modification indices in the final model (item codes follow the domain-sequential numbering shown in Fig 3; Access = AC1-AC3, AC5-AC13, i.e., AC4 and AC14 dropped), 10 met both the modification-index threshold and a genuine item-content overlap on review of the item wording; the remaining 15 lacked a defensible theoretical rationale and were not retained (Table 3).

The four-point Likert-type response format (Very Difficult to Very Easy) used in this study follows the same format adopted in developing the HLS-EU-Q [22], the foundational instrument informing our conceptual framework similarly analyzed this scale using standard factor analytic and reliability methods, without polychoric correlations or WLSMV estimation. Following this established precedent, the present study applied standard exploratory and confirmatory factor analysis methods accordingly.

## Results

### Demographic characteristics of samples

There were 1,235 respondents, of whom 46.2% were women, 26.1% were in their third year, 63.7% identified as Buddhist, and 39.7% resided in the northeast region (Table 1).

**Table 1:** Demographic characteristics of undergraduates in Thailand (n = 1,235).

Demographic characteristics	Number	Percentage (%)
Gender		
Cis- Male	378	30.60
Cis- Female	570	46.17
LGBTQ+	287	23.23
Educational level		
1^st^ year	233	18.86
2^nd^ year	311	25.18
3^rd^ year	322	26.07
4^th^ year	237	19.19
5^th^ year	73	5.91
6^th^ year	59	4.79
Religion		
Buddhism	787	63.72
Christianity	172	13.92
Islam	158	12.79
Other	118	9.57
Region		
Central	245	19.83
North	287	23.23
Northeast	490	39.67
South	213	17.27

### Exploratory factor analysis (EFA)

Exploratory factor analysis (EFA) was performed using principal axis factoring with Promax rotation. The suitability of the data for factor analysis was supported by the Kaiser Meyer Olkin (KMO) measure of sampling adequacy (KMO = 0.950) and Bartlett’s test of sphericity (χ² = 40,122.987, df = 1,176, p < 0.001). The inspection of the scree plot revealed a distinct elbow after the fourth factor, supporting the retention of a four factor solution (Fig 2). Two items with factor loadings below 0.30 were removed, in a final instrument comprising 47 items. The four factor solution explained 53.17% of the total variance (Table 2). Parallel analysis (Horn’s method, 1,000 replications) supported only 3 factors unambiguously: the first three eigenvalues (20.85, 2.93, 2.41) exceeded the 95th-percentile threshold, while the fourth (1.657) fell just below it (1.715). Four factors were retained primarily on a priori theoretical grounds (the four domain SHL framework) rather than the scree plot alone. Table 2’s eigenvalues and percentage-of-variance are initial (total-variance) values, not PAF common-variance extraction sums. Cross-loading items, including those with primary/secondary loadings differing by less than 0.11, were retained based on theoretical fit to their assigned domain rather than a strict simple-structure rule.

**Fig 2:**
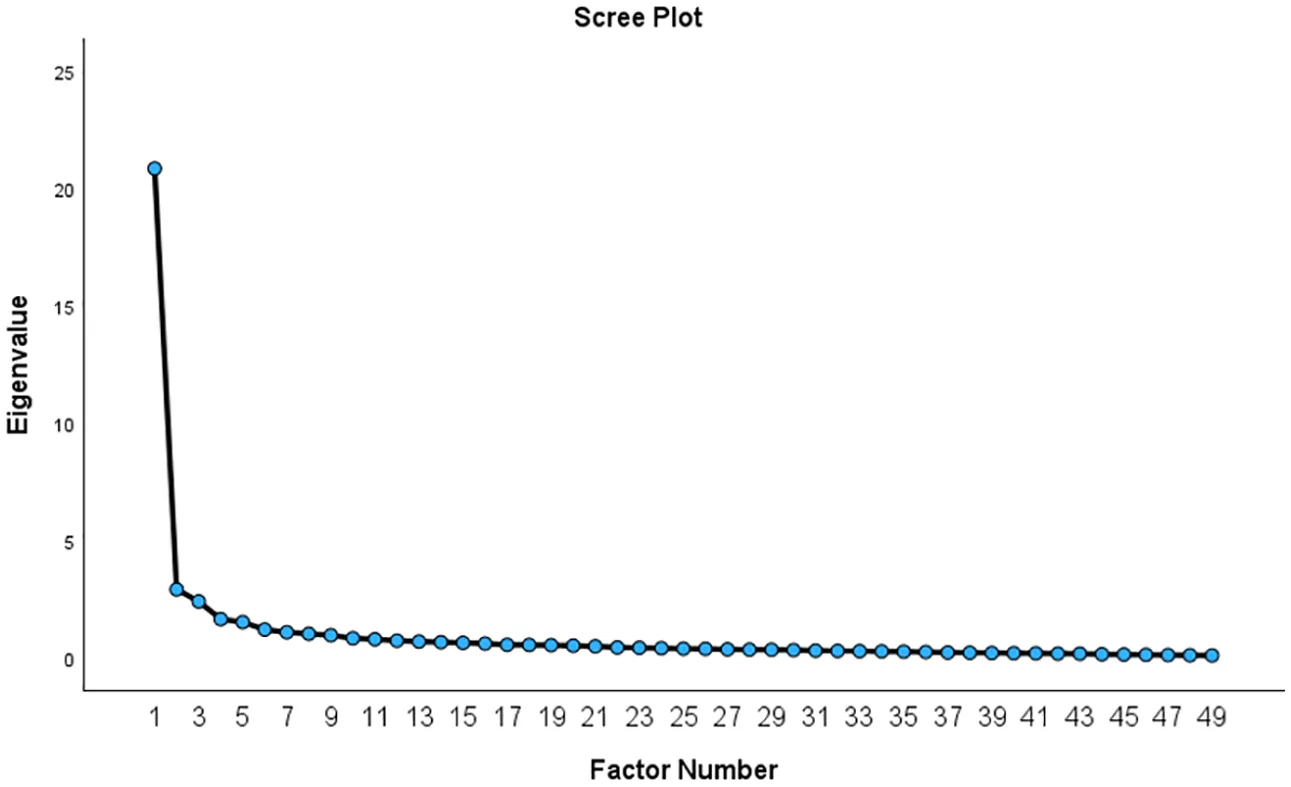
Scree test criterion. Scree plot of eigenvalues by factor number (n = 300). The elbow after the fourth factor supported retention of a four-factor solution.

**Table 2:** Results were obtained from an exploratory factor analysis of the sexual health literacy measurement tool for undergraduate students (SHL-US) (n = 300).

Item	Domain code	Component				Status
		1	2	3	4	
Item1	AC1	–	–	–	–	Retained
Item2	AC2	0.445				Retained
Item3	AC3	0.363				Retained
Item4	AC4	0.385				Dropped before CFA
Item5	AC5	0.561				Retained
Item6	AC6	0.795				Retained
Item7	AC7	0.845				Retained
Item8	AC8	0.764				Retained
Item9	AC9	0.575				Retained
Item10	AC10	0.837				Retained
Item11	AC11	0.368		0.311		Retained
Item12	AC12	0.537				Retained
Item13	AC13	0.541				Retained
Item14	AC14	0.567				Dropped before CFA
Item15	UN1		0.717			Retained
Item16	UN2		0.483			Retained
Item17	UN3		0.674			Retained
Item18	UN4		0.627			Retained
Item19	UN5		0.659			Retained
Item20	UN6		0.692			Retained
Item21	UN7		0.689			Retained
Item22	UN8		0.721			Retained
Item23	UN9		0.578			Retained
Item24	UN10		0.530			Retained
Item25	UN11		0.738			Retained
Item26	UN12		0.832			Retained
Item27	APR1				0.806	Dropped before CFA
Item28	APR2			0.593		Retained
Item29	APR3			0.758		Retained
Item30	APR4			0.732		Retained
Item31	APR5			0.836		Retained
Item32	APR6			0.834		Retained
Item33	APR7			0.770		Retained
Item34	APR8			0.836		Retained
Item35	APR9			0.906		Retained
Item36	APR10			0.831		Retained
Item37	APR11			0.755		Retained
Item38	APR12				0.709	Dropped before CFA
Item39	APL1	–	–	–	–	Retained
Item40	APL2			0.580	0.373	Retained
Item41	APL3				0.794	Retained
Item42	APL4				0.469	Retained
Item43	APL5				0.535	Retained
Item44	APL6				0.496	Retained
Item45	APL7				0.660	Retained
Item46	APL8				0.554	Retained
Item47	APL9	0.387			0.493	Retained
Item48	APL10	0.408			0.499	Retained
Item49	APL11	0.444			0.491	Retained
**Eigenvalue**		20.403	2.48	1.976	1.188	
**Variance (%)**		41.639	5.074	4.044	2.425	
**Total variance (%)**		41.639	46.713	50.746	53.171	

### Confirmatory factor analysis (CFA)

To identify the most appropriate measurement structure for the Sexual Health Literacy (SHL) scale, three alternative CFA models were evaluated: 1) four-correlated-factors model, 2) first-order CFA model, and 3) second-order CFA model. The comparative goodness-of-fit indices are presented in Table 3. The results indicate that the final second-order model (45 items) provided the best overall model fit. Model was shown in Fig 3. The initial second-order model estimated with 47 items showed suboptimal fit (Table 3). Inspection of standardized factor loadings identified two items with loadings below 0.50; these were removed, and re-estimation of the second-order model with the remaining 45 items yielded a substantially improved fit. The results provided a good fit to the data for four dimensions within the 45 items: access to information (12 items), understanding (12 items), appraisal (10 items), and application (11 items). The confirmatory model (45 items, 10 content-justified correlated residuals) gave: χ² = 4150.084, df = 931, χ²/df = 4.458, GFI = 0.841, AGFI = 0.823, CFI = 0.914, TLI = 0.908, RMSEA = 0.055, NFI = 0.892 (standardized RMR = 0.023).

**Table 3:** Comparison of goodness-of-fit indices for confirmatory factor analysis models.

Model	χ²/df	CFI	TLI	GFI	AGFI	RMR	RMSEA
Four-correlated (47 items, no correlated residuals)	6.49	0.862	0.855	0.798	0.778	0.173	0.069
Second-order, a priori (45 items, no correlated residuals)	5.019	0.899	0.894	0.906	0.796	0.025	0.059
Unidimensional/ one-factor (47 items)	6.102	0.873	0.865	0.815	0.795	0.172	0.066
Second-order, final (45 items, 10 content-justified correlated residuals)	4.458	0.914	0.908	0.841	0.823	0.023	0.055

CFI, comparative fit index; TLI, Tucker Lewis index; GFI, goodness of fit index; AGFI, adjusted goodness-of-fit index; RMR, root of mean square residual; RMSEA, root mean square error of approximation.

**Fig 3:**
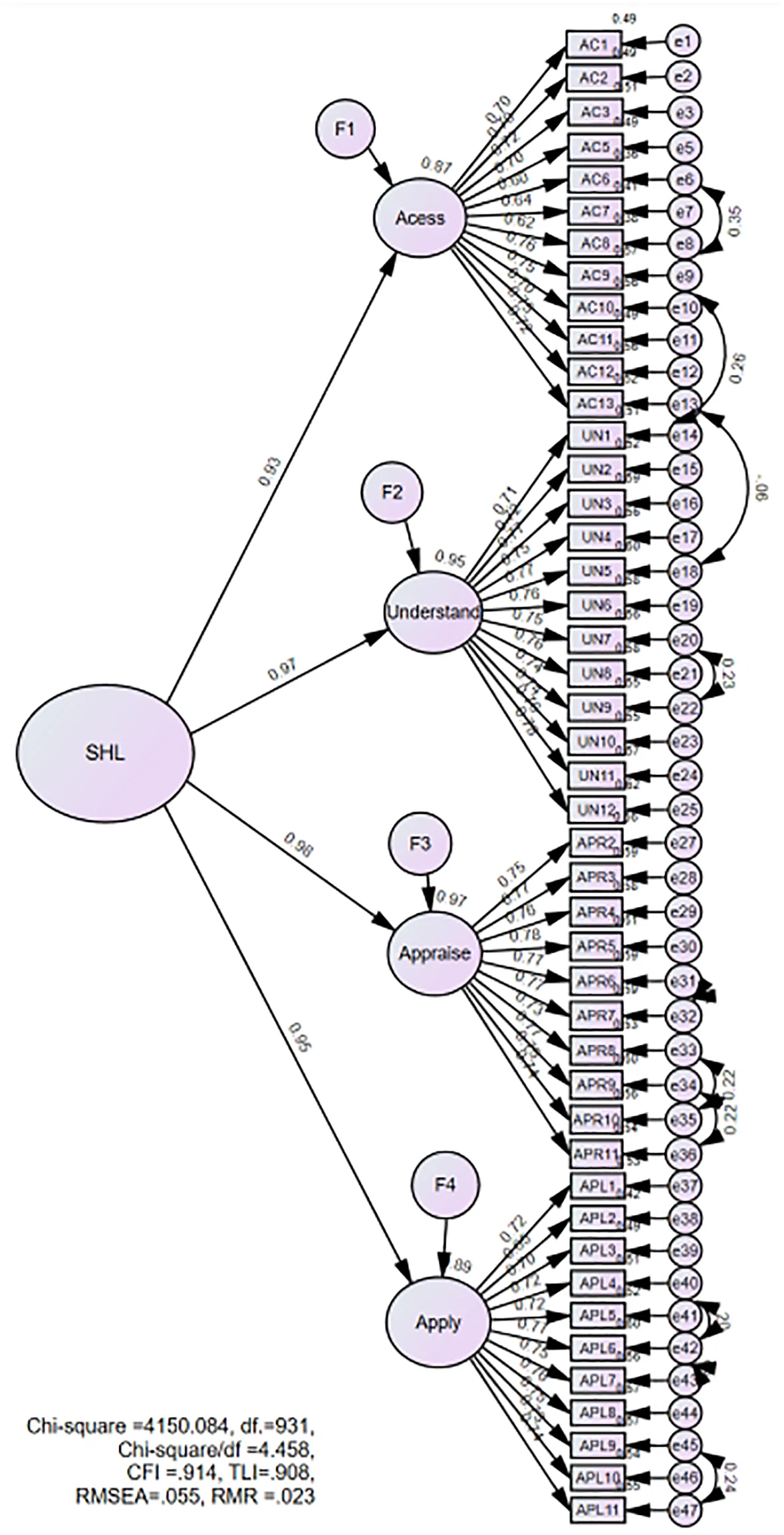
Result of the confirmatory factor analysis of the Sexual Health Literacy Tool for undergraduates in Thailand. Second-order confirmatory factor analysis model (n = 1,235). Sexual health literacy (SHL) is modeled as a higher-order factor reflected by four first-order factors: Access (AC1-AC3, AC5-AC13; 12 items), Understanding (UN1-UN12), Appraisal (APR2-APR11), and Application (APL1-APL11; 11 items). Values shown are standardized factor loadings (all p < 0.001); individual values on correlated-residual arcs are not shown.

### Convergent validity

Applying the 0.50 AVE threshold set in Methods, convergent validity was supported for three of the four dimensions (Understanding, Appraisal, Application); the Accessing dimension’s AVE (0.486) fell just below this threshold. The questionnaire’s dimensions showed convergent validity based on standardized factor loadings (λ), which ranged 0.599–0.757 (Accessing), 0.715–0.785 (Understanding), 0.727–0.781 (Appraisal), and 0.648–0.773 (Application). Composite reliability (CR) ranged from 0.919 (Accessing) to 0.940 (Understanding), and average variance extracted (AVE) ranged from 0.486 (Accessing) to 0.575 (Appraisal) (Table 4).

**Table 4:** The Sexual Health Literacy Assessment Tool for undergraduate students has standardized factor loadings (λ), composite reliability (CR), and average variance extracted (AVE).

Sexual health literacy	λ	CR	AVE
Accessing	0.599-0.757	0.919	0.486
Understanding	0.715−0.785	0.940	0.565
Appraise	0.727-0.781	0.931	0.575
Applying	0.648-0.773	0.926	0.532

All component weights have p-values of < 0.001.

**λ**, standardized factor loadings; CR, composite reliability; AVE, average variance extracted.

### Discriminant validity

Discriminant validity was evaluated using the Fornell-Larcker criterion and the HTMT ratio (same CFA solution, n = 1,235). The square root of each domain’s AVE (0.697–0.758) was lower than the inter-domain correlations (0.884–0.956), and HTMT ratios (0.868–0.959) exceeded conventional thresholds for all domain pairs; both indicate the four domains function as strongly correlated facets of a single general SHL factor rather than as fully distinct competencies. A chi-square-difference test supported this structure: the four-correlated-factor model fit significantly better than a one-factor alternative (delta-chi-square = 1631.5, delta-df = 16, p < .001), indicating the four-domain structure is best interpreted and scored as one overall higher-order SHL score, with domain sub-scores treated as correlated indicators rather than independent subscales. Internal consistency was strong across domains (Cronbach’s alpha 0.901–0.937; item-total correlations 0.303–0.750, all meeting the 0.30 criterion). Because composite reliability (Table 4) assumes uncorrelated residuals, McDonald’s omega was also computed accounting for the 10 correlated residuals (Access 0.910, Understanding 0.935, Appraisal 0.924, Application 0.931), confirming reliability consistent with the CR values.

### Reliability

Forty-five SHL-US items were assessed for reliability by estimating Cronbach’s alpha coefficient. The results showed that all the factors had satisfactory internal consistency. The Cronbach’s alpha coefficient for the overall scale was 0.976, and the Item-Total Correlation was above 0.300 (range: 0.284–0.755).

## Discussion

Sexual health literacy (SHL) is the ability and skill to care for one’s own sexual health. The instrument developed to measure undergraduate SHL in Thailand comprised 45 items. After reviewing the literature, the researchers developed a framework of questions covering SHL and defined each field’s domain. The four parts of the SHL-US are 1) information access, 2) information understanding, 3) information appraisal, and 4) information application. Items including promoting sexual health, preventing unsafe sexual behavior, and sexual health were the focus of the questions. To generate 4-component questions aligned with the development of the 47-item European Health Literacy Scale for adults [22], variables were synthesized during the data collection phase to ensure they were comprehensive and relevant. The content validity index was 0.94 at both the item and the scale level, which meets the accepted standard. This approach helped to ensure consistent content and instruments [40]. The reliability analysis of the questions found a corrected item-total correlation value between 0.284 and 0.755, meaning that each question item is related to the entire questionnaire, and the appropriate value should be > 0.3 [39]. The internal consistency analysis revealed that Cronbach’s alpha value of the entire version was an acceptable 0.976, showing that the items in each component are related [26]. Exploratory factor analysis identified components that explained 53.17% of the total variance, with each component comprising more than three items; following [38], which stipulates a minimum of three items per component. Confirmatory factor analysis supported the four-component structure, though fit was more modest than initial indices suggested once only content-justified correlated residuals are retained (CFI = 0.914), and the four domains did not meet Fornell-Larcker or HTMT discriminant-validity criteria (inter-domain correlations 0.88–0.96); the domains are better characterized as strongly correlated facets of one general SHL factor than as fully distinct competencies, and the second-order (hierarchical) scoring approach used in this study is preferable to treating the four domain scores as independent subscales. Although the present instrument adapted its items from the European Health Literacy Scale for Adults (HLS-EU-Q47) [22], that questionnaire addresses general health rather than sexual health specifically, and was not developed for undergraduate populations. In the Thai context, Manwong et al. surveyed 730 Grade 8 students in a rural eastern province during the COVID-19 period and found that 76.3% scored at a low-to-moderate SHL level, with SHL emerging as the strongest correlate of pregnancy- and STD-preventive behaviour in multivariable analysis (b = 0.119, 95% CI 0.08–0.16, p < 0.001) [3]. That measure, however, was validated through item-objective congruence and internal consistency alone, so its four-domain structure was assumed rather than empirically recovered; the present study contributes the structural evidence that prior Thai work has lacked. Existing SHL instruments likewise reflect narrower scopes. In Thailand, the SHL-PPA was developed for adolescents aged 13–19 years and conceptualises SHL specifically in relation to pregnancy prevention, with all three of its empirically derived components framed around that single outcome; its validation relied on exploratory factor analysis, and the developers noted that the tool is not a gold-standard measure of adolescent SHL and that further psychometric examination is warranted [12]. For convergent comparison, the four-domain structure obtained here aligns with the independently developed Sexual Health Literacy for Adults (SHELA) questionnaire, which likewise resolved into four dimensions (access; reading and understanding; evaluation and analysis; application) and whose structural validity and reliability have since been examined in an Iranian sample [11]. SHELA was not, however, a source for item generation in the present study, whose items were adapted solely from the HLS-EU-Q47 [22]; the structural convergence of two instruments developed independently in different cultural settings therefore strengthens, rather than duplicates, the case for the four-domain conceptualization.

## Conclusion

The SHL-US (45 items) demonstrated an acceptable four-factor structure, convergent validity for three of the four domains, and satisfactory internal consistency among Thai undergraduate students. The four domains are best interpreted as correlated facets of a single overall SHL construct rather than as fully independent subscales, and the instrument is recommended for use primarily as a single overall SHL score. Suggestions for future research include: 1) applying this tool in further studies with appropriate psychometric follow-up, including criterion and test-retest validation; 2) developing sexual health promotion programs informed by SHL-US findings; and 3) conducting longitudinal measurement to track changes in sexual health literacy over time.

### Strengths and limitations

The SHL-US, a 45-item four-domain tool, showed good validity and reliability among Thai undergraduate students, supporting its value as an initial measure in this population. Several aspects warrant consideration in future use. Sampling followed a multi-stage design (institution, faculty, classroom) but was limited to two institutions per region with voluntary participation; clustering was not modeled in estimation, and participation counts were not reported. Ordinal-item estimation used Pearson covariances rather than polychoric correlations with WLSMV, which may modestly attenuate loadings. While 23.23% of respondents identified as LGBTQ + , item content leans heteronormative, and the Gender variable combines cisgender status, sex, and orientation, limiting invariance and differential-item-functioning analyses; how the STI/pregnancy-history exclusion criterion was ascertained under anonymous administration is unclear and may have affected LGBTQ+ respondents disproportionately. Test-retest reliability, criterion/concurrent/predictive/known-groups validity, and a social desirability measure were outside this study’s scope. The cross-sectional design does not support causal or longitudinal inference, and the scoring and standard-setting procedures would benefit from fuller specification in future work.

## Supporting information

S1 Questionnaire Sexual Health Literacy Measurement Tool among Undergraduate Students (SHL-US). The questionnaire used in this study to measure sexual health literacy among undergraduate students.
(DOC)

S1 Dataset De-identified dataset used in the psychometric validation of the Sexual Health Literacy Measurement Tool among Undergraduate Students (SHL-US) (n = 1,235). (XLSX)
