# Supplementary material for: Development and psychometric validation of the Sexual Health Literacy Measurement Tool for Undergraduate Students (SHL-US) in Thailand
Source: PLoS One. 2026 Oct 9;21(10):e0359881. doi: 10.1371/journal.pone.0359881 (PMC13652164; doi:10.1371/journal.pone.0359881)
Supplement: Supplementary file 1 [file pone.0359881.s001.doc]

**Sexual Health Literacy Measurement Tool among Undergraduate Students** **(SHL-US)**

**Instruction:** Please mark ✓ in the box that most accurately reflects your opinion.

| **Item** | **How easy or difficult is it for you to…** | **Perceived Difficulty** | | | |
| --- | --- | --- | --- | --- | --- |
|  |  | **Very Difficult** | **Quite Difficult** | **Easy** | **Very Easy** |
| **Accessing** | | | | | |
| 1 | Search for information sources on your own when you need information about methods to prevent sexually transmitted diseases and unwanted pregnancies. |  |  |  |  |
| 2 | Search for information about vaccinations and screening for sexually transmitted diseases. |  |  |  |  |
| 3 | Search for desired and reliable sources of sexual health information, such as hospitals, sexual health clinics, etc. |  |  |  |  |
| 4 | Search for desired and reliable sources of sexual health care information by inquiring from parents, teachers, experts, etc. |  |  |  |  |
| 5 | Search for sex-related hotlines, such as 1323 (Sexual Problem Consultation Hotline), when you have sexual problems. |  |  |  |  |
| 6 | Visit a sexual health service provider every time there is a problem. |  |  |  |  |
| 7 | Search for sexual health services within educational institutions. |  |  |  |  |
| 8 | Search for information about activities to promote sexual behavior, such as exercise, relaxation, healthy eating, etc. |  |  |  |  |
| 9 | Search for reliable information about how to prevent STDs and unwanted pregnancy so you can recommend or share it with your friends. |  |  |  |  |
| 10 | Search out for appropriate sexual health care. |  |  |  |  |
| 11 | Search out how to prevent sexually transmitted diseases and unwanted pregnancy. |  |  |  |  |
| 12 | Search out about the dangers and effects of unsafe sexual behavior. |  |  |  |  |
| **Understanding** | | | | | |
| 13 | Understand how to prevent sexually transmitted diseases and unwanted pregnancy by learning from reliable sources. |  |  |  |  |
| 14 | Understand when reading manuals or documents about information on how to prevent sexually transmitted diseases and unwanted pregnancy from the Internet, magazines, television, etc. |  |  |  |  |
| 15 | Understand why you should get the HPV vaccine to protect against sexually transmitted diseases. |  |  |  |  |
| 16 | Understand why you should be screened for STDs |  |  |  |  |
| 17 | Understand easily explanations from sexual health providers or educational guides about how to promote sexual health and how to prevent risky sexual behavior. |  |  |  |  |
| 18 | Understand that the best and safest way to prevent unwanted pregnancy and STDs is to use a condom. |  |  |  |  |
| 19 | Explain information about promoting appropriate sexual behavior and how to prevent risky sexual behavior to friends or people around them so that they can understand. |  |  |  |  |
| 20 | Understand the rationale behind sexual health warnings from government campaigns on safe sex and STD prevention. |  |  |  |  |
| 21 | Understand information about promoting appropriate sexual behavior and preventing risky sexual behavior presented in symbols such as pictures, words, and signs in various places. |  |  |  |  |
| 22 | Understand that STD screening or HPV vaccination is a way to prevent STDs. |  |  |  |  |
| 23 | Understand the risks and effects of unsafe sexual behavior. |  |  |  |  |
| 24 | Understand what to do when you have sexual health problems. |  |  |  |  |
| **Appraise** | | | | | |
| 25 | Compare the advantages and disadvantages before deciding to believe information from any source about how to prevent sexually transmitted diseases and unwanted pregnancy. |  |  |  |  |
| 26 | Verify the accuracy of information and sources before believing it when receiving new information. |  |  |  |  |
| 27 | Consult a trusted person and do your research before deciding to trust them immediately if you have any doubts or questions about how to prevent STDs and unwanted pregnancy. |  |  |  |  |
| 28 | Study and compare information from multiple sources to confirm your correct understanding. |  |  |  |  |
| 29 | Know how to find information about STD prevention and unwanted pregnancy to verify the accuracy of the information before believing and acting on it. |  |  |  |  |
| 30 | Consult specialists regarding the accuracy of practices and information supporting appropriate sexual behavior and methods for preventing risky sexual behavior. |  |  |  |  |
| 31 | Verify the source of the information before believing or sharing it when new sexual health information and services become available. |  |  |  |  |
| 32 | Analyze the root causes of the problem and find several solutions to help you decide what to do. |  |  |  |  |
| 33 | Compare information from multiple sources before believing or following it. |  |  |  |  |
| 34 | Consider your options for appropriate methods to prevent unwanted pregnancy and sexually transmitted diseases on your own. |  |  |  |  |
| **Applying** | | | | | |
| 35 | Follow the advice of your teacher, specialist, or sexual health provider to promote and prevent unsafe sexual behavior. |  |  |  |  |
| 36 | Refusal of sexual intercourse that involves oral sex |  |  |  |  |
| 37 | Use a condom every time before having sex. |  |  |  |  |
| 38 | Follow the directions on the product packaging (e.g., birth control pills, emergency contraception, and condoms). |  |  |  |  |
| 39 | Screening or vaccination to prevent sexually transmitted diseases |  |  |  |  |
| 40 | Share information with your family, friends, or relatives when you receive advice on how to prevent sexually transmitted diseases and unwanted pregnancy. |  |  |  |  |
| 41 | Refrain from having sex if the sex is unprotected, such as using a condom. |  |  |  |  |
| 42 | Avoid situations that are high risk of sexually transmitted infections and unwanted pregnancy, such as going to nightlife, drinking alcohol, having multiple partners, etc. |  |  |  |  |
| 43 | Recognize unsafe sexual situations and be able to plan to avoid unsafe sexual behaviors. |  |  |  |  |
| 44 | Always check the expiration date and damage and tear of the condom before use. |  |  |  |  |
| 45 | Practice proper and consistent practices to prevent sexually transmitted diseases and unwanted pregnancy. |  |  |  |  |
